# Social Reward Behaviour in Two Groups of European Grey Wolves (*Canis lupus lupus*)—A Case Study

**DOI:** 10.3390/ani13050872

**Published:** 2023-02-27

**Authors:** Hana Tebelmann, Udo Gansloßer

**Affiliations:** Institute of Zoology and Evolutionary Research with Phyletic Museum, Friedrich Schiller University Jena, 07743 Jena, Germany

**Keywords:** social reward, wolves, prosociality, mammals, social behaviour, *Canis lupus lupus*, novel object, behavioural chains

## Abstract

**Simple Summary:**

Prosocial behaviour is shown by a variety of mammals. Prosocial behaviours can also be part of social feedback mechanisms. For group-living mammals, group coordination and group cohesion are crucial factors for survival. Therefore, group-living mammals display a broad variety of social behaviours towards their group members. Canidae are known to be socially organised mammals. Wolves are one of the most cooperative and social canine species. In this case study, we investigated social feedback mechanisms in two European grey wolf groups. The social feedback was observed after novel object interaction, bold behaviour, and the individual behaviours of single individuals. The purpose of this paper is to focus on prosocial behaviour that might serve as social reward. We assume that there is social reward behaviour as a category that falls within social feedback in grey wolves.

**Abstract:**

Prosociality occurs in many species and is likely to be a crucial factor for the survival of group-living animals. Social feedback is an important mechanism for the coordination of group decisions. Since group-living animals with specific personality axes, i.e., boldness, are known to provide certain benefits for their group, bold actions might receive more prosocial feedback than other actions. Our case study aims to determine whether bold behaviour, i.e., novel object interaction (Nobj), might be answered more frequently with prosocial behaviours. We investigated the differences in the frequency of occurrence in prosocial behaviours after three different individual actions in two groups of grey wolves. We aim to outline the development of a social reward behavioural category as part of social feedback mechanisms. We used Markov chain models for probability analyses, and a non-parametric ANOVA to test for differences between the influences of individual behaviours on the probability of a prosocial behaviour chain. We additionally tested for the potential influences of age, sex and personality on the frequency of Nobj. Our results suggest that bold interactions are more often responded to with prosocial behaviour. Bold behaviour might be more often socially rewarded because of its benefits for group-living animals. More research is needed to investigate whether bold behaviour is more frequently responded to prosocially, and to investigate the social reward phenomenon.

## 1. Introduction

Personality traits, which are consistent inter-individual differences in behaviour within populations, are a widespread phenomenon across various animal species [1,2]. Studies have shown that mammals [3], birds [4], fish [5,6], arthropods [7,8,9,10,11], amphibians [12] and cephalopods [13] exhibit consistent individual differences in behavioural traits, which are expressed in specific situations [14]. Extraversion [15,16] and boldness [16,17,18,19,20] are two of the most well-known phenomena in animal personality research. Boldness describes a consistent difference between individuals in their response to perceived risk [1]. Boldness is considered to be part of a major ‘proactive–reactive’ axis of personality variation, where boldness is one of a suite of behaviours which includes exploration, activity and aggression, which correlate positively with each other [12,21]. When presented with a startling stimulus or a novel object, individuals may differ consistently in their responses over repeated observations, a behavioural complex that might influence species dispersal as well as the approach to obtaining food in a task involving a novel object. Bolder individuals might have greater food intake when foraging [22], or take more risks to acquire food, investigate novel objects, or explore new environments. Previous research has shown that bolder individuals may be more likely to lead [23,24,25], whereas shy individuals may be more likely to group [26] and to respond to the decisions of their bolder conspecifics [27,28,29]. As bolder individuals are more prone to be neophilic and to take risks, they potentially are beneficial for the rest of their group, as they might serve as foragers, investigators, guardians or warners, since each individual profits from such division of labour.

Across taxa, individuals show the cognitive ability to use their observations of the social interactions of others to inform their own behaviours, including several primate species [30,31], ravens [32], hyenas [33] and fish [34,35]. While it has been shown that individuals observe each other and react to these observations, we do not currently understand how individuals integrate this information on the outcomes of their own interactions with observations of others’ interactions, on their future actions. Social feedback, the tendency of a group to answer prosocially or agonistically to another group member’s individual behaviour, is an important mechanism for the coordination of group decisions. We suggest that there might be a special type of social feedback: social reward. We define social reward as prosocial behaviours that are shown in direct response to a desirable and group-beneficial behaviour, i.e., bold behaviour, and aim to encourage the behaviour. This reward mechanism could be partly intended in order to increase the frequency of the occurrence of the displayed behaviour, or to encourage other group members to engage in the behaviour. Bold behaviours may be risky for individuals, but they may also prove to be beneficial to the group, and might therefore be supported by social feedback. In distinction to prosocial behaviour, which in addition to confirmation, has various other functions such as reproduction, rank verification, group cohesion, togetherness etc., we define social reward behaviour as a behavioural category that exclusively serves positive social feedback and is thus part of the social feedback mechanism. Social reward, as prosocial behaviours sent by the group towards an individual of the group to give feedback on a previous action, could be a special tool to encourage the actions of a group member that are in the interest of the group but that cannot be carried out by other group members, such as actions that require boldness. Social feedback or social reward might be useful within groups to engage in group-benefiting actions.

Emotional contagion, the transmission of emotional states from one animal to another, might also be involved in social feedback processes and group decision-making. Most studies on emotional contagion in non-human animals have focused on the transmission of negative emotional states, e.g., fear, but observations on farm animals suggest that animals may transmit both negative and positive emotions: respectively, pleasant and unpleasant emotions such as joy or fear [36,37]. Pigs, for example, responded differently to being reunited with group mates who had experienced either a negative or a positive treatment. They exhibited decreased activity and exploration when group mates received a negative treatment, and increased social contact and exploration when group mates received a positive treatment [37]. Since bolder animals, such as fast-bold explorers, show a reduced stress-induced glucocorticoid release compared to slow-shy [38], prosocial acts towards bold-behaving group members could also be influenced by mechanisms of emotional contagion.

Here, we take a look at the relationship between bold actions and social feedback mechanisms in two groups of European grey wolves (*Canis lupus lupus*), to address the question of how bold behaviours can influence the frequency of prosocial interactions towards the bold individual. We observed two groups of European grey wolves in two different zoological facilities in Germany. The observations were part of a large-scale project on cooperative behaviour in canids, which also included Arctic wolves, Hudson Bay wolves and Timber wolves, as well as various other canids.

## 2. Materials and Methods

### 2.1. Study Animals

Our case study included two different wolf groups, with 5 individuals per group, at Zoo Wingst and Schwarze Berge Wildlife Park (see Table 1).

### 2.2. Novel Object

**Figure 1:**
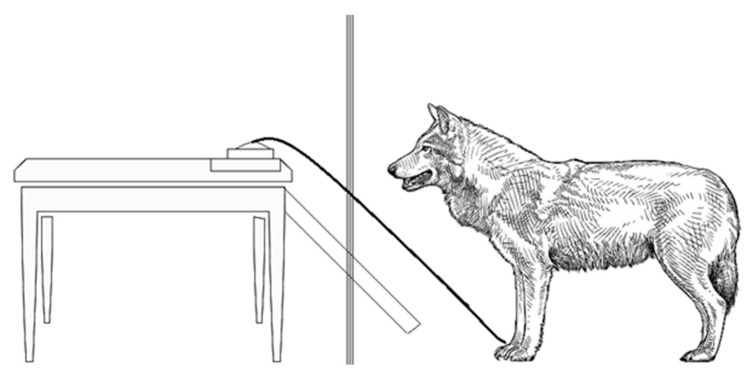
Exemplary presentation of the novel object and the described experimental set-up, adapted from Stefan_Alphonso. Stock Illustration ID:1397963840.

An apparatus of 1.80 m × 0.60 m × 1.20 m was installed in front of the enclosure (see Figure 1). The purpose of the apparatus was to study foraging cooperation in Canidae; therefore, it was equipped with food tubes, ropes for pulling and food flaps that were connected to the ropes (see Supplementary Materials Figures S1 and S2). The European grey wolf groups did not use the apparatus communally, and no cooperative food procurement was shown.

### 2.3. Observational Methodology

**Figure 2:**
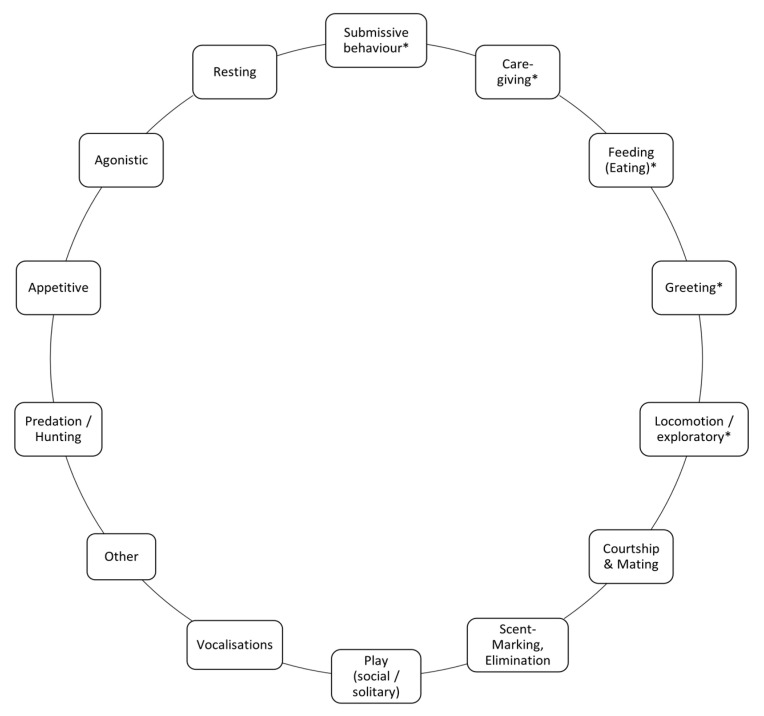
Modified version of [40] on wolf behaviour. The asterisk indicates the categorisation of “behaviours of interest” into behavioural categories.

Behavioural observations were made in total for 192 h from June to August 2020, and from May to June 2021, via means of a rare event (“all-occurrence”) sampling [39], which led to 242 behavioural observations and the record of 140 behaviours of interest in total. We used the ethogram of Goodmann et al. (2002) [40] to place the behaviours that we observed to a behavioural category (see Table 2). For a detailed version of the ethogram, see [40]. The behaviours of interest are categorised under submissive behaviour, care-giving behaviour, locomotion/exploratory behaviour and feeding (see Figure 2).

#### Definitions of Behaviours of Interest

We define behaviours of interest, referring to those behaviours as initial behaviours (see Table 3) that have led to a social reward behaviour chain (see Table 4). The social reward behaviour chain was recorded as such if a subsequent element directly (<15 s) followed the previous behaviour.

Initial behaviours commonly led to a complete or partially performed behavioural chain, which included the behavioural states listed in Table 4. Behavioural states that were performed with the same frequency and that fell under the same behavioural category in our ethogram (see Figure 2), were classified as a combined state.

### 2.4. Markov Chain Modelling

Markov chains quantify the dependence of an event on preceding events [41,42] or an on initial state. There are several degrees of dependence. If sequencing events are independent, they are described by a zero-order Markov chain. In the case where an event depends only on the immediately preceding one, it fits a first-order Markov chain. If an event depends on the two most preceding events, it is a second-order Markov chain, and so on. We decided to assess the difference in transition from one event to another, and the probability to reach the succeeding state of the behavioural chain, depending on the initial state of the observed sequence. To simplify the analytical design, we concentrated only on a first-order Markov chain model. Transition probabilities (from initial to preceding to succeeding behaviours) were determined in all three chains using:

(1) $$pij=\frac{aij}{\sum_{j=1}^{6}aij},\overset{6}{\underset{j=1}{\sum }}pij=1$$

where *i* is the initial behaviour, *j* is the succeeding behaviour (*i* and *j* range from 1 to 6, because there are six behavioural states in the chain), aij is the number of transitions observed from behaviour *i* to *j*, and *pij* is the transition probability from *i* to *j* in the Markov chain. Since the initial state influenced the succeeding state, we used an initial state vector of [0, 1]. We then calculated the steady-state vector (v) for our transition matrix. To determine the state of the system after one step (A1), the following applies: A1 = P − A0. P is a transfer matrix; it can be used to describe how a system changes over time from an initial-state vector A0. To determine the state of the system after one step (A1), the following applies: A1 = P − A0. Thus, to calculate any step t + 1, it only needs the transfer matrix P and the vector in the previous step At + 1. To be exact: At + 1 = P − At.

Using Python version 2022.2.4 (PyCharm Community Edition), we have calculated any number of steps by multiplying the vector of the current step by the transfer matrix P to reach the next step. At some point of $P_{n\;}$, the vector no longer changes with the steps, and thus remains the same after each step, and so v = P − v is true.

### 2.5. Statistical Evaluation of Markov Chain Probabilities

The results of the Markov chains were used to determine whether there were differences in the influence of the initial state on the success of the behavioural chain. We used a non-parametric ANOVA, the Kruskal–Wallis test, to determine whether differences between initial states existed. The influence on the initial state of a successful behavioural chain (reaching the succeeding state) were calculated using the R version 4.2.2. (R Development Core Team, R Foundation for Statistical Computing, Vienna, Austria), with the “ggpubr” package for Windows. Normality was assessed using the Kolmogorov–Smirnov test. The relationship between the initial state and the success score was estimated using the Kruskal–Wallis test. To compare variables with significant inter-group variability, the Wilcoxon rank-sum was used for data with non-normal normal distribution, and the Dunn’s test for pairwise comparisons to test for significant means. The returned *p*-values were adjusted using Bonferroni corrections for multiple comparisons. The results were presented as a median. The level of statistical significance was preset to *p* < 0.05.

### 2.6. Influence Factors on the Frequency of Novel Object Interaction

Factors such as age, sex and personality traits are known to influence behaviour. Therefore, we tested the influences of age, sex and the personality traits of the individuals on the frequency of novel object interaction.

To determine whether there was a statistically significant mean difference in the frequency of novel object interaction between female and male European grey wolves, we ran an independent samples t-test using package “ggpubr” in R. To estimate the relationship between the age of the individuals and the frequency of novel object interaction, a Wilcoxon rank sum test was performed using the package “coin”. We estimated the data of 5 female and 5 male European grey wolves from 2 different groups. For age differences in novel object interaction, we compared the age data of the same groups, creating two different age groups (mean 2.5 | 9).

In order to determine the influence of personality factor on the frequency of novel object interaction, the DOGS questionnaire and the Monash Canine Personality Questionnaire—Revised (MCPQ-R) were given to the animal keepers of each group. We adapted the DOGS questionnaire [43] and the MCPQ-R for canids living in zoological institutions, and modified the questions accordingly (see Supplementary Materials). The DOGS personality questionnaire [43] includes the factors of trainability, sociability, extraversion and calmness. The MCPQ-R [44] contains the factors extraversion, self-assuredness/motivation, training focus, amicability and neuroticism. Since the personality factors of the questionnaires are similar, we grouped the factors extraversion/extraversion and sociability/amicability into personality dimensions. Since the trainability factor contains items of playfulness and intelligence, and is comparable to the Big Five dimension of Openness [45], we split this factor into two factors: Curiosity/Openness and Playfulness. Curiosity refers to an interest in new things, perceptiveness, learnability and cognitive flexibility. Playfulness refers to social play with conspecifics, playful interest in people and object-related play (see Table 5).

We also calculated a David’s Score (DS) [46] as an alternative method to access dominance rank. DS is a type of cardinal rank (dominance rank is an ordinal type of ranking) that is calculated from an individual’s proportion of wins and losses in relation to the wins and losses of its opponents; ranging from −3 to 3 for triads, where −3 represents a maximum proportion of losses and 3 represents a maximum proportion of wins [47]. The DS was combined with the personality traits as a possible influencing factor on the frequency of novel object interaction. As per Ley et al. [44] and Turcsán et al. [43], the raw scores for each adjective within each personality factor subscale were summed and divided by the maximum score possible for the subscale. The result was converted to a percentage, thereby creating a percentage score for each of the five personality factors for every individual. To evaluate the influence of the personality factors and DS on the frequency of novel object interaction, a correlation matrix was created, and a multiple correlation analysis was performed. The multiple correlation analysis was carried out using the package “Hmsic”. A linear regression analysis was then performed using the packages “dplyr”, “broom” and ggpubr”.

## 3. Results

### 3.1. Observations

Despite the animals’ neophobia and object-related fear, four individual group members showed repeated approaches and exploratory behaviours towards the novel object (max. 16, min. 1). Some group members—two in one group, three, partially, in another—independently approached the novel object directly and interacted with it by sniffing on it or pulling the rope located in the enclosure. There was only one animal at a time approaching the apparatus while the others kept distance, with a maximum of 14 interactions of a single individual in one group, and 16 interactions of another individual in the other group. After pulling, the animals immediately left the vicinity of the novel object and returned to their group. When returning to the group, the animals that interacted directly with the apparatus were welcomed by the group, while the animals that were in the close-up range as observers were not greeting, but they contributed to group formation. The “interactors’” muzzles were licked, they were greeted with a tail wag, the muzzle was licked by every other group member, and submissive behaviour was shown, after which the group retreated in a body. This behavioural chain was shown after each direct approach to the novel object, and was uniform in sequence in both wolf groups. This behavioural chain reinforced and increased the approach behaviour to the novel object, but did not reduce object-related fear sufficiently enough for observations of foraging cooperation. In one group, these behaviours seemed to result in another animal occasionally approaching the apparatus independently, which was then greeted in the same way, but were ultimately prevented from doing so by the “first interactor”.

Behavioural chain:

Initial (A0) → group formation (A1) → greet. and sub. gest. (A2, A3, A4, A5) → group leaving together (A6).

### 3.2. Markov Probabilities of Initial Behaviours on the Succeeding Behaviours

**Figure 3:**
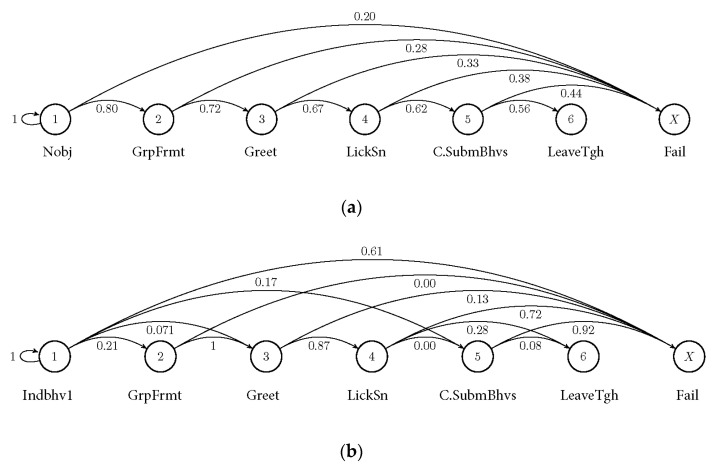

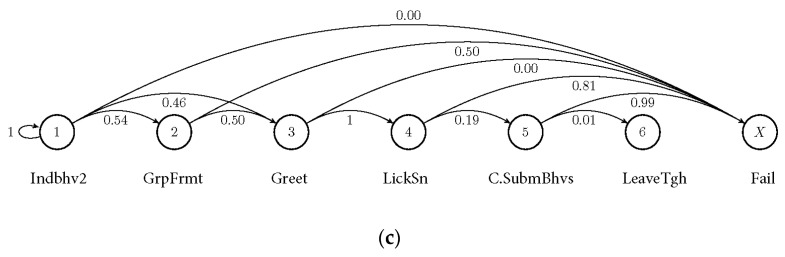
Markov chains representing the probabilities of transition in behavioural state: (**a**) behavioural chain after novel object interaction; (**b**) behavioural chain after individual behaviour “run”; (**c**) behavioural chain after individual behaviour “forage”. Only transitions within the behavioural chain are represented; values are percentages. Behavioural states are defined in Table 4. Initial behaviours are defined in Table 3.

When the behavioural chain started with the initial state Nobj (Novel object interaction), the probability to reach the succeeding state was 56% after seven steps, which was the same as after 24 steps (vector [0.56, 0.44]). Starting with individual behaviours, the calculated probability of reaching the succeeding state was 08% for Indbhv1 and 01% for Indbhv2 after seven steps (see Figure 3), and remaining steady after 8 and 24 steps, respectively. Success (A6) was defined as the completion of the behavioural chain, while failure (fail) was defined as the probability of the termination or non-completion of the behavioural chain; for example, if the chain stops at an any state before (A6). The relationship between the initial state and the success score was 0.63 for Nobj (Novel object interaction), 0.264 for Indbhv1 (run), and 0.266 for Indbhv2 (forage) (see Table 6).

Since the data consist of a behavioural chain where states can merge into each other, and since states can be omitted, but succeeding states cannot merge into preceding states, it is represented as a probability chain and not a probability matrix.

**Figure 4:**
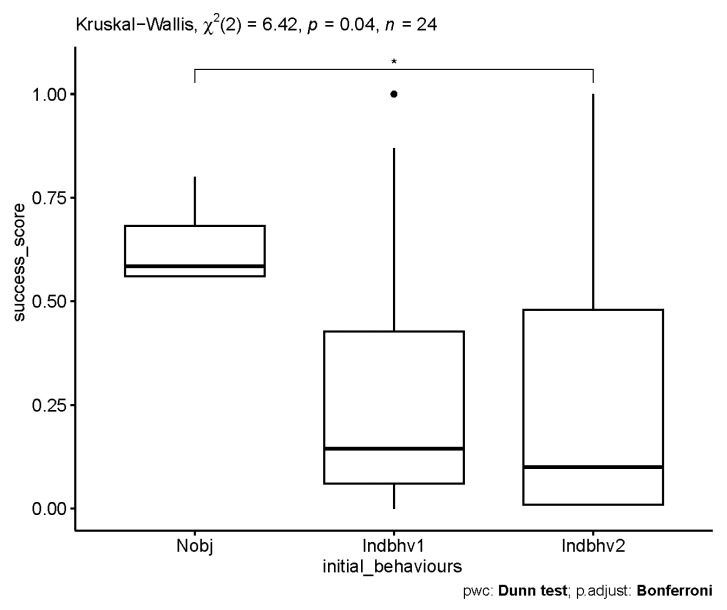
Effect of initial state interactions on transitions in behavioural state of the behavioural chain, based on differences in transition probabilities (pij(Nobj) − pij(Indbhv1) − pij(Indbhv2). The star symbol indicates the statistically significant values, the dot symbol indicates the outliers.

The difference between the initial states were only statistically significant for Nobj and Indhbv2; the statistical significance was *p* = 0.015 before and *p* = 0.04 after Bonferroni correction; for Indbhv1 and Indbhv2 = *p* = 1, for Nobj and Indbhv1 = *p* = 0.20 after Bonferroni corrections (see Figure 4).

### 3.3. Influence of Sex, Age and Personality Traits on the Frequency of Novel Object Interaction

**Figure 5:**
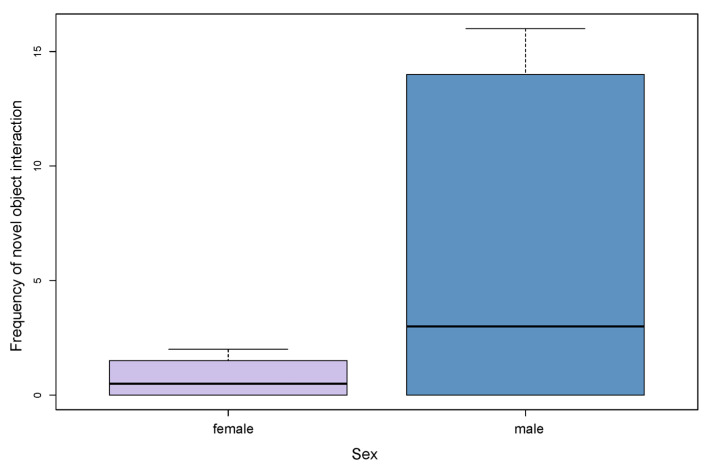
Influence of sex on frequency of novel object interaction.

The results of the relationship between sex and novel object interaction were (0.75 ± 0.957) for female wolves compared to the male group (6.60 ± 7.797). There was no statistically relevant difference between the frequency of novel object interaction in male and female wolves (*t* = 1.04, *df* = 8, *p* = 0.18) (see Figure 5).

**Figure 6:**
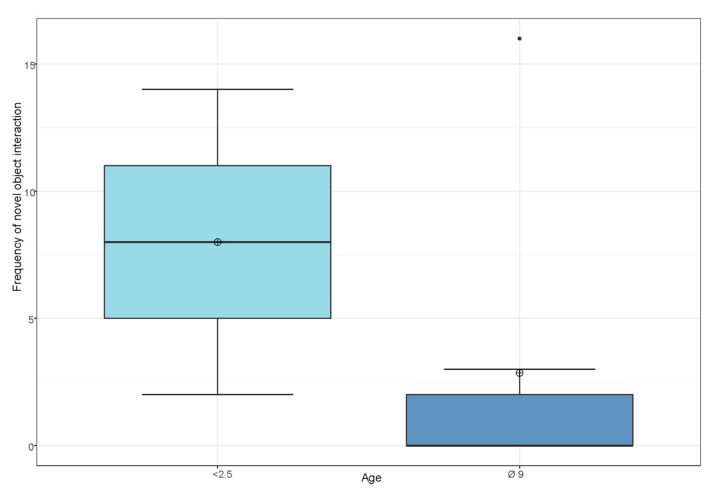
Influence of age on frequency of novel object interaction. The dot symbol indicates the outliers.

For age differences in novel object interaction, we compared the age data of the same groups, creating two different age groups (mean 2.5 | 9). We found no effect of age on frequency of novel object interaction (*W* = 11, *Z* = 1.22, *p* = 0.33) (see Figure 6).

Personality factor means were relatively low with high standard derivation, suggesting that wolves may generally have lower scores in behavioural traits such as playfulness or motivation, or they may score lower in these categories (see Table 7). In particular, the scores for playfulness can be explained, as wolves do not exhibit paedomorphism. We performed a multiple t-test to test for the influence of sex and age on each personality dimension, no statistically relevant results were found.

**Figure 7:**
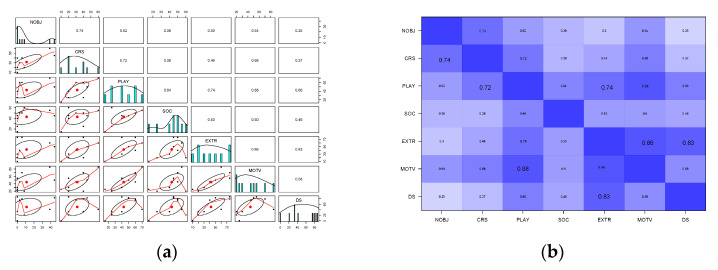
Correlation matrix of personality traits, DS and frequency of novel object interaction. (**a**) Correlation plot pairs for multiple correlations, (**b**) Correlation plot matrix.

All personality traits showed a positive correlation with the frequency of novel object interaction, but this effect was only statistically significant for the factor “curiosity/openness” (CRS) (*r* = 0.73, *p* = 0.02). All other dimensions or factors showed a positive trend, which was marginal. The other two factors with a higher correlation to frequency of novel object interaction (NOBJ) were “playfulness” (PLAY) (*r* = 0.52, *p* = 0.15) and “motivation” (MOTV) (*r* = 0.54, *p* = 0.12). The lowest correlation was found in David’s Score, the “Dominance Score” (DS) (*r* = 0.25, *p* = 0.51) (see Figure 7a).

Some dimensions and factors also showed positive correlations with each other. For example, the two traits “Curiosity” and “Playfulness”, which can be assigned to the factor “Trainability”, are positively correlated (*r* = 0.72, *p* = 0.28). “Motivation” and “Playfulness” also show a significant positive correlation (*r* = 0.87, *p* = 0.02). “Extraversion” (EXTR) is positively correlated to “Playfulness” (*r* = 0.74, *p* = 0.02), “Motivation” (*r* = 0.86, *p* = 0.003) and “Dominance Score” (*r* = 0.82, *p* = 0.006). “Sociability” was most positively correlated to “playfulness” (*r* = 0.64, *p* = 0.06) (see Figure 7b).

**Figure 8:**
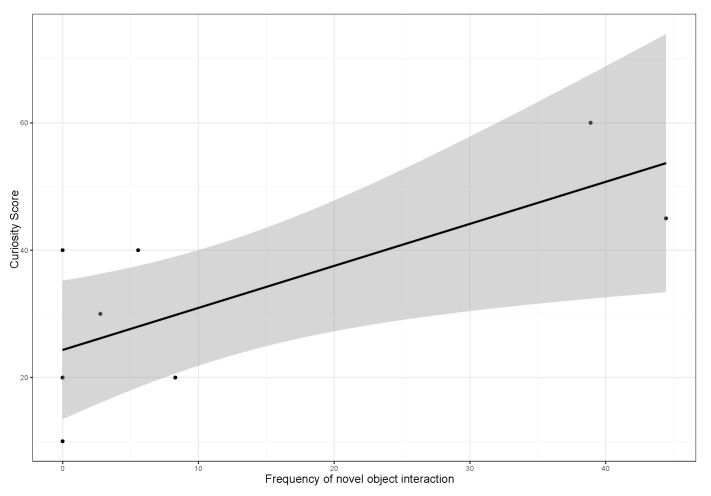
Linear regression model of influence of curiosity on frequency of novel object interaction with the grey area as 95% confidence level. The dots indicate the respective data points. Data points outside the grey confidence level are outliers.

Due to the statistically significant positive correlation between “curiosity” and the frequency of novel object interaction (Nobj), we also conducted a linear regression analysis (see Figure 8). The results show a moderate correlation between the personality factor “curiosity” and the frequency of novel object interaction (*R*^2^ = 0.48, *df* = 8, *p* = 0.02).

## 4. Discussion

The application of transition matrix analyses to the study of behaviour provided more information than standard techniques would have; therefore, it proved to be useful as a tool for the probability analysis of behavioural chains. As animals are not infinite-state automata, Markov analyses might have limitations. We have to take into account that with a transition matrix, where every state can change into any other state of the matrix, different probabilities would probably have been obtained than with our behaviour chain, in which transitions only run in one direction. In addition, we excluded other possible initial behaviours that never led to completion: for example, success, of the behavioural chain, in order not to potentially drive up the significance level of the results. As we started collecting the data based on the observed chain of behaviour, a bias can be assumed. Behavioural states are generally difficult to sample adequately in the field without observer bias; therefore, we categorised the observed behaviours to established categories in the reliable [40] ethogram to avoid possible miscategorisation or strong assumptions about the functions of the observed behaviours. Since many of the behaviours we observed are part of the [40] ethogram, we consider categorisation errors and interpretation errors to be, for the most part, unlikely. Our results are mainly not statistically significant, which may be due to the sample size, as well as the excluded data. Nevertheless, our results show that the influence of bold behaviour, such as an interaction with a novel object, more often led to a behavioural chain of prosocial behaviours, whereas other individual behaviours that are also attention-grabbing, such as running around or foraging, did not regularly lead to the success of such a behavioural chain. Despite the low statistical power of the results, we would like to point out the relevance of the findings, as social reward is an unmentioned concept so far, but our results provide first indications that this behavioural category might exist as a subcategory of social feedback. Wolves are known to be one of the most cooperative canine species. Likely, the cooperative propensity is derived from the fact that each individual needs its other group members for survival. The group functions as a unit in which each individual collaborates in territory defence, hunting and the rearing of offspring [48]. One example for bold behaviour benefiting the group is cooperation during intergroup conflicts. Individuals actively dealing with conflicts for the benefit of the group, regardless of possible disadvantages for themselves, provide an example of a cooperative behaviour that is costly to participants, because it involves a considerable expenditure of energy and risk of injury, and that often results in benefits to both the cooperating and noncooperating group members, in terms of increased access to contested resources [49]. Individuals with more affiliative partners are more likely to act boldly in social contexts [50]. It could be assumed that not only the already existing number of social affiliations, but also the expected feedback in terms of social prestige might play a role in decision-making and action processes. Expected social prestige could promote risky, bold behaviour. In addition, pleasant emotional states might result from successful risk taking, e.g., caused by a dopaminergic reward response. The likeliness of the social reward-chain after novel object interaction could also be partly explained by social support, which plays a role when group members are exposed to stress [51]. Social support, which can express through seeking contact or rapprochement, as well as affiliative behaviour, can have a stress-reducing effect [52,53]. Therefore, calming mechanisms may also have played a role as an answer to the novel object interaction, given that such was potentially stressful for generally neophobic animals such as wolves. However, due to the lower stress response in risky or novel situations that bold individuals generally show [38], emotional contagion may also have played a role, in that group members who felt stressed by the sight of the new object, but then perceived the approach of a bold group member, felt less stressed and thus also showed a positive response to the approach. This could also have been a factor in the social reward response. Thus, a combination of different social behaviours and mechanisms may well play a role; both the reinforcement of bold behaviour and the reduction in stress on the individual and group levels.

Yet, it is known that boldness has several advantages on the group-level [49,54], but also some on the individual level, including that bold individuals often have a comparatively high status in the group, as aggressiveness, exploratory behaviour and boldness are positively correlated across individuals [16,54,55]. In our results, extraversion and dominance score were positively correlated. Since extraversion is associated with boldness, as the shyness–boldness continuum shares traits with the extraversion–introversion axis [56,57], it is not surprising that individuals that scored higher in extraversion also had the higher status within their group, such as the highest success in conflicts. This also supports the hypothesis that boldness is positively related to status. Although boldness is generally age-dependent—as has been shown in dogs (Canis lupus familiaris) [58]—as well as sex-dependent [58,59,60,61], our results did not show a significant influence of age or sex on bold behaviour, i.e., the frequency of novel object interaction. Possible explanations are that wolves might be less variable in their personality or behavioural traits, as with dingoes [62], but the results are likely also influenced by the small sample size, as well as the large age gaps between the wolves.

It is difficult to observe prosocial behaviours that follow bold individual behaviours in observations of wild animals, or even in observations of the social behaviours of captive animals, without a special experimental set-up. Therefore, it is likely that social reward is more frequently exhibited in wolves, as well as mammals in general, but it may be categorised as prosocial individual behaviours. It is likely that social reward does not only occur as a result of bold actions, but refers to diverse behaviours that can be beneficial to the group, such as cooperative behaviours. To better understand animal behaviour, it is important to be able to identify it as accurately and as precisely as possible. Therefore, we suggest that the phenomenon of social feedback should be investigated in more detail, and potential subcategories such as social reward should be explored. If we can identify reward mechanisms in group processes, this may prove helpful in assessing the animals’ individual and group-oriented decisions, their social ecology, and possibly also the development of group dynamics. This can be useful, for example, in assessing which animal might take which future rank in captive animal groups, to detect conflicts between captive group-living animals at an early stage, or to contribute to the understanding of the processes and the adaptive mechanisms of wild animals. By identifying behaviour that is worth rewarding, it might even be possible to draw conclusions about evolutionary drivers.

As social reward and social-reward chains are not an established concept, we suggest that this potential phenomenon should be addressed, and that social reward is worth considering as part of prosociality. Due to our small sample size, more research is needed, with larger group sizes and in wider contexts, to investigate social reward. However, we hypothesise that social reward mechanisms exist among animals, or wolves, and that they can contribute to behavioural reinforcement, particularly in the area of group decision-making and the benefits of individual actions to groups or populations.

As our findings are part of a case study, the data are limited. Until more research is conducted, our results should be treated as tentative.

## 5. Conclusions

Our findings suggest that there may be specific behavioural chains as part of social feedback in grey wolves. The prosocial behaviours of those behavioural chains could have reward functions, which is potentially due to the benefits of an individual’s bold actions for the group. Our results may be interpreted to indicate that bold behaviour in social mammals, such as wolves, is responded to with social reward and prosocial behaviour, which is potentially via reason of the benefits that individual bold actions can implicate for the group.

Due to the small number of groups and the potential bias in the observation, our results should be interpreted with caution. Further research is needed to investigate social feedback mechanisms in wolves and other social animals.

## Figures and Tables

**Table 1:** Animals included in the study.

Name	Abbreviation	Sex	Birth Date	Facility
Runa	EU1	Female	2018	Schwarze Berge
Yuuki	EU2	Male	2018	Schwarze Berge
Dunja	EU3	Female	2013	Schwarze Berge
Django	EU4	Male	2014	Schwarze Berge
Skadi	EU5	Female	2017	Schwarze Berge
Wolfgang	EUW1	Male	2011	Zoo Wingst
Rudolf	EUW2	Male	2011	Zoo Wingst
Anfa	EUW3	Female	2011	Zoo Wingst
Wolle	EUW4	Male	2013	Zoo Wingst
Andra	EUW5	Female	2011	Zoo Wingst

**Table 2:** Modified ethogram [40]. Behaviours and categories added to the ethogram are written in bold.

Behavioural Category	Original Category [40]	Subcategories	Behaviours in the Social Reward Chain
Agonistic behaviours	Agonistic behaviours	Elicited Aggression Food-related Aggression Sex-related Aggression All-Out Attack Defence and Submission Offensive Threat Ritualised Attack, Counterattack, Fight	None
Play behaviours	Play behaviours	Agonistic Play Social Play Solitary Play	None
Caregiving	Caregiving, Care Solicitation	None	Approach Sniffle Face Wipe Follow Hold Out Face, Airplane Ears
**Submissive behaviours**	Not existent	None	**Expose Belly** **Down and Lick** **Lick Snout** **Low Posture** Follow
Feeding (Eating)	Feeding (EAT)	None	Approach Cache Carry Object Drag Eat **Forage** Grab Lick Mouth Paw Tug
Greeting	Greeting	None	Airplane Ears Approach **Body Rub** Ears Back Ears Pricked Greet **Group Formation/Group Together** Grin Hug Hum Lick **Leave Together** Parallel Gait Parallel Walk Sniff Noses Tail Wag
Locomotion/exploratory	Locomotion	None	Amble Approach Avoid Follow Observation **Object Interaction** Observation Jump **Run**
Scent-Marking, Elimination	Scent-Marking, Elimination	None	None
Other	Other	None	Explore Indirect Approach Orient Wander
Predation, Hunting	Predation, Hunting	None	None
Resting	Resting	None	None

**Table 3:** Definitions of initial behaviours.

Initial Behaviours	Description of Observed Behaviours
Novel object interaction (Nobj)	Approaching the novel object (apparatus) up to maximum 1 m distance, and sniffing on the rope or interacting with it by trying to pull the rope/pulling the rope
Forage	Walking around the enclosure, searching for leftovers of food or dead animals, eating, or hiding food
Run	Running around individually in the enclosure from one area to another

**Table 4:** Definitions of the social reward behaviour states in a behavioural chain.

State	Description of Observed Behaviours
Group formation (GrpFrmt)	Individuals grouping together, observing the individual performing an “initial behaviour”
Greeting (Greet)	Tail wagging, body rubbing and face/body sniffing of group formation towards “active performer”
Submissive behaviour (LickSn)	Snout licking, lowered body posture, whimpering, lying on back/exposing belly towards the individual performing the “initial behaviour”
Combined submissive behaviours (C.SubmBhv)	Body rubbing, ears back, opening group formation and including the individual performing the “initial behaviour” to the group
Leaving together (LeaveTgh)	Leaving proximity of “initial behaviour performance location” as a united group

**Table 5:** Questions and items related to personality factors curiosity and playfulness in [43,44].

Factor	Questions and Items to Determine Factor Values
Curiosity/Openness	“is inventive and resourceful when it comes to finding or reaching hidden food or toys” “does not have many interests apart from eating and sleeping, e.g., is unexplorative, uncurious, show limited interest in new objects, humans or animals” “has a good grasp of things and learns quickly” “attentive” “intelligent” “clever”
Playfulness	“is enthusiastic and encourages his peers to play”
	“is easy to get excited about new play ideas”
	“often does not understand what is being asked of him in play situations”

**Table 6:** Initial state and success score of Markov chain calculation (*n* = 24).

Initial State	Success Score (Mean ± SD)
Nobj (novel object interaction)	0.63 * ± 0.091
Indhbhv1 (“run”)	0.318 ± 0.394
Indbhv2 (“forage”)	0.279 ± 0.362

* = statistically significant value.

**Table 7:** Mean values for personality factors of the two European wolf groups.

Personality Trait/Factor	Result (Mean ± SD)
Playfulness	31.66 ± 15.81
Extraversion	42.22 ± 24.25
Curiosity	42.77 ± 18.72
Sociability	40.75 ± 14.38

## Data Availability

The data obtained during the case study are available upon request.

## Supplementary Materials

The following supporting information can be downloaded at: https://www.mdpi.com/article/10.3390/ani13050872/s1, Figure S1: Technical drawing of the apparatus and its function; Figure S2: Photo of the apparatus in use on another wolf group.

## References

[1] Réale D., Reader S.M., Sol D., McDougall P.T., Dingemanse N.J. (2007). Integrating Animal Temperament within Ecology and Evolution. Biol. Rev..

[2] Bell A.M., Hankison S.J., Laskowski K.L. (2009). The Repeatability of Behaviour: A Meta-Analysis. Anim. Behav..

[3] Réale D., Martin J., Coltman D.W., Poissant J., Festa-Bianchet M. (2009). Male Personality, Life-history Strategies and Reproductive Success in a Promiscuous Mammal. J. Evol. Biol..

[4] Groothuis T.G.G., Claudio C. (2005). Avian personalities: Characterization and epigenesis. Neuroscience and biobehavioral reviews. Neurosci. Biobehav. Rev..

[5] Wilson A.D.M., Stevens E.D. (2005). Consistency in Context-Specific Measures of Shyness and Boldness in Rainbow Trout, Oncorhynchus Mykiss. Ethology.

[6] Ariyomo T.O., Watt P.J. (2012). The Effect of Variation in Boldness and Aggressiveness on the Reproductive Success of Zebrafish. Anim. Behav..

[7] Reaney L.T., Backwell P.R.Y. (2007). Risk-Taking Behavior Predicts Aggression and Mating Success in a Fiddler Crab. Behav. Ecol..

[8] Mowles S.L., Cotton P.A., Briffa M. (2012). Consistent Crustaceans: The Identification of Stable Behavioural Syndromes in Hermit Crabs. Behav. Ecol. Sociobiol..

[9] Toscano B.J., Gownaris N.J., Heerhartz S.M., Monaco C.J. (2016). Personality, Foraging Behavior and Specialization: Integrating Behavioral and Food Web Ecology at the Individual Level. Oecologia.

[10] Bridger D., Bonner S.J., Briffa M. (2015). Individual Quality and Personality: Bolder Males Are Less Fecund in the Hermit Crab *Pagurus bernhardus*. Proc. R. Soc. B Biol. Sci..

[11] Belgrad B.A., Karan J., Griffen B.D. (2017). Individual Personality Associated with Interactions between Physiological Condition and the Environment. Anim. Behav..

[12] Sih A., Bell A., Johnson J.C. (2004). Behavioral Syndromes: An Ecological and Evolutionary Overview. Trends Ecol. Evol..

[13] Sinn D.L., Gosling S.D., Moltschaniwskyj N.A. (2008). Development of Shy/Bold Behaviour in Squid: Context-Specific Phenotypes Associated with Developmental Plasticity. Anim. Behav..

[14] Réale D., Gallant B.Y., Leblanc M., Festa-Bianchet M. (2000). Consistency of Temperament in Bighorn Ewes and Correlates with Behaviour and Life History. Anim. Behav..

[15] Gosling S.D., John O.P. (1999). Personality Dimensions in Nonhuman Animals. Curr. Dir. Psychol. Sci..

[16] Finkemeier M.-A., Langbein J., Puppe B. (2018). Personality Research in Mammalian Farm Animals: Concepts, Measures, and Relationship to Welfare. Front. Vet. Sci..

[17] Chapman B.B., Hulthén K., Blomqvist D.R., Hansson L.-A., Nilsson J.-Å., Brodersen J., Anders Nilsson P., Skov C., Brönmark C. (2011). To Boldly Go: Individual Differences in Boldness Influence Migratory Tendency. Ecol. Lett..

[18] Dammhahn M., Almeling L. (2012). Is Risk Taking during Foraging a Personality Trait? A Field Test for Cross-Context Consistency in Boldness. Anim. Behav..

[19] Beckmann C., Biro P.A. (2013). On the Validity of a Single (Boldness) Assay in Personality Research. Ethology.

[20] Herde A., Eccard J.A. (2013). Consistency in Boldness, Activity and Exploration at Different Stages of Life. BMC Ecol..

[21] Bevan P.A., Gosetto I., Jenkins E.R., Barnes I., Ioannou C.C. (2018). Regulation between Personality Traits: Individual Social Tendencies Modulate Whether Boldness and Leadership Are Correlated. Proc. R. Soc. B Biol. Sci..

[22] McDonald N.D., Rands S.A., Hill F., Elder C., Ioannou C.C. (2016). Consensus and Experience Trump Leadership, Suppressing Individual Personality during Social Foraging. Sci. Adv..

[23] Biro P.A., Stamps J.A. (2008). Are Animal Personality Traits Linked to Life-History Productivity?. Trends Ecol. Evol..

[24] Jolles J.W., Ostojić L., Clayton N.S. (2013). Dominance, Pair Bonds and Boldness Determine Social-Foraging Tactics in Rooks, Corvus Frugilegus. Anim. Behav..

[25] Kurvers R.H.J.M., Krause J., Croft D.P., Wilson A.D.M., Wolf M. (2014). The Evolutionary and Ecological Consequences of Animal Social Networks: Emerging Issues. Trends Ecol. Evol..

[26] Bell A.M., Sih A. (2007). Exposure to Predation Generates Personality in Threespined Sticklebacks (*Gasterosteus Aculeatus*). Ecol. Lett..

[27] Krause J., James R., Croft D.P. (2010). Personality in the Context of Social Networks. Philos. Trans. R. Soc. B Biol. Sci..

[28] Harcourt J.L., Biau S., Johnstone R., Manica A. (2010). Boldness and Information Use in Three-Spined Sticklebacks. Ethology.

[29] Pike T.W., Samanta M., Lindström J., Royle N.J. (2008). Behavioural Phenotype Affects Social Interactions in an Animal Network. Proc. R. Soc. B Biol. Sci..

[30] Bergman T.J., Sheenan M.J. (2013). Social Knowledge and Signals in Primates. Am. J. Primatol..

[31] Wittig R.M., Crockford C., Langergraber K.E., Zuberbühler K. (2014). Triadic Social Interactions Operate across Time: A Field Experiment with Wild Chimpanzees. Proc. R. Soc. B Biol. Sci..

[32] Massen J.J.M., Pašukonis A., Schmidt J., Bugnyar T. (2014). Ravens Notice Dominance Reversals among Conspecifics within and Outside Their Social Group. Nat. Commun..

[33] Engh A.L., Siebert E.R., Greenberg D.A., Holekamp K.E. (2005). Patterns of Alliance Formation and Postconflict Aggression Indicate Spotted Hyaenas Recognize Third-Party Relationships. Anim. Behav..

[34] Chase I.D., Tovey C., Spangler-Martin D., Manfredonia M. (2002). Individual Differences versus Social Dynamics in the Formation of Animal Dominance Hierarchies. Proc. Natl. Acad. Sci. USA.

[35] Earley R.L., Dugatkin L.A. (2002). Eavesdropping on Visual Cues in Green Swordtail (*Xiphophorus Helleri*) Fights: A Case for Networking. Proc. R. Soc. London. Ser. B Biol. Sci..

[36] Reimert I., Bolhuis J.E., Kemp B., Rodenburg T.B. (2015). Emotions on the Loose: Emotional Contagion and the Role of Oxytocin in Pigs. Anim. Cogn..

[37] Reimert I., Fong S., Rodenburg T.B., Bolhuis J.E. (2017). Emotional States and Emotional Contagion in Pigs after Exposure to a Positive and Negative Treatment. Appl. Anim. Behav. Sci..

[38] Taborsky B., English S., Fawcett T.W., Kuijper B., Leimar O., McNamara J.M., Ruuskanen S., Sandi C. (2021). Towards an Evolutionary Theory of Stress Responses. Trends Ecol. Evol..

[39] Mann J. (1999). Behavioral Sampling Methods for Cetaceans.: A Rewie and Critique. Mar. Mammal Sci..

[40] Goodmann P.A.K.E., Wolf Ethogram W.J., Eckhard H. (2002). Ethology Series No. 3.

[41] Guttorp P. (2018). Stochastic Modeling of Scientific Data.

[42] Caswell (2001). Matrix Population Models.

[43] Turcsán B., Kubinyi E., Miklósi Á. (2011). Trainability and boldness traits differ between dog breed clusters based on conventional breed categories and genetic relatedness. Appl. Anim. Behav. Sci..

[44] Ley J., Bennett P., Coleman G. (2009). A refinement and validation of the Monash Canine Personality Questionnaire (MCPQ). Appl. Anim. Behav. Sci..

[45] Draper T.W. (1995). Canine analogs of human personality factors. J. Gen. Psychol..

[46] de Vries H. (1998). Finding a dominance order most consistent with a linear hierarchy: A new procedure and review. Anim. Behav..

[47] Gammell M.P., de Vries H., Jennings D.J., Carlin C.M., Hayden T.J. (2003). David’s score: A more appropriate dominance ranking method than Clutton-Brock et al.’s index. Anim. Behav..

[48] Cordoni G., Palagi E. (2019). Back to the Future: A Glance Over Wolf Social Behavior to Understand Dog–Human Relationship. Animals.

[49] Nunn C.L., Deaner R.O. (2004). Patterns of Participation and Free Riding in Territorial Conflicts among Ringtailed Lemurs (*Lemur Catta*). Behav. Ecol. Sociobiol..

[50] Bonanni R., Valsecchi P., Natoli E. (2010). Pattern of Individual Participation and Cheating in Conflicts between Groups of Free-Ranging Dogs. Anim. Behav..

[51] Rault J.-L. (2012). Friends with Benefits: Social Support and Its Relevance for Farm Animal Welfare. Appl. Anim. Behav. Sci..

[52] Beery A.K., Kaufer D. (2015). Stress, Social Behavior, and Resilience: Insights from Rodents. Neurobiol. Stress..

[53] Panksepp J.B., Jochman K.A., Kim J.U., Koy J.J., Wilson E.D., Chen Q., Wilson C.R., Lahvis G.P. (2007). Affiliative behavior, ultrasonic communication and social reward are influenced by genetic variation in adolescent mice. PLoS ONE.

[54] Wilson A.D., Godin J.-G.J. (2009). Boldness and Behavioral Syndromes in the Bluegill Sunfish, Lepomis Macrochirus. Behav. Ecol..

[55] Dahlbom S.J., Lagman D., Lundstedt-Enkel K., Sundström L.F., Winberg S. (2011). Boldness Predicts Social Status in Zebrafish (*Danio Rerio*). PLoS ONE.

[56] Gosling S.D. (2001). From mice to men: What can we learn about personality from animal research?. APA PsycNet.

[57] Walker D.L. (2020). Extraversion—Introversion. The Wiley Encyclopedia of Personality and Individual Differences.

[58] Kubinyi E., Turcsán B., Miklósi Á. (2009). Dog and owner demographic characteristics and dog personality trait associations. Behav. Process..

[59] Øverli Ø., Sorensen C., Nilsson G.E. (2006). Behavioral indicators of stress-coping style in rainbow trout: Do males and females react differently to novelty?. Psysiol. Behav..

[60] Schuett W., Dall S. (2009). Sex differences, social context and personality in zebra finches, Taeniopygia guttata. Anim. Behav..

[61] Ward-Fear G., Brown G.P., Pearson D.J., West A., Rollins L.A., Shine R. (2018). The ecological and life history correlates of boldness in free-ranging lizards. Ecosphere.

[62] Smith B.P. (2014). Living with Wild Dogs: Personality Dimensions in Captive Dingoes (*Canis Dingo*) and Implications for Ownership. Anthrozoos.

