# Supplementary figures and images for: Social Reward Behaviour in Two Groups of European Grey Wolves (Canis lupus lupus)—A Case Study
Source: Animals (Basel). 2023 Feb 27;13(5):872. doi: 10.3390/ani13050872 (PMC10000159; doi:10.3390/ani13050872)

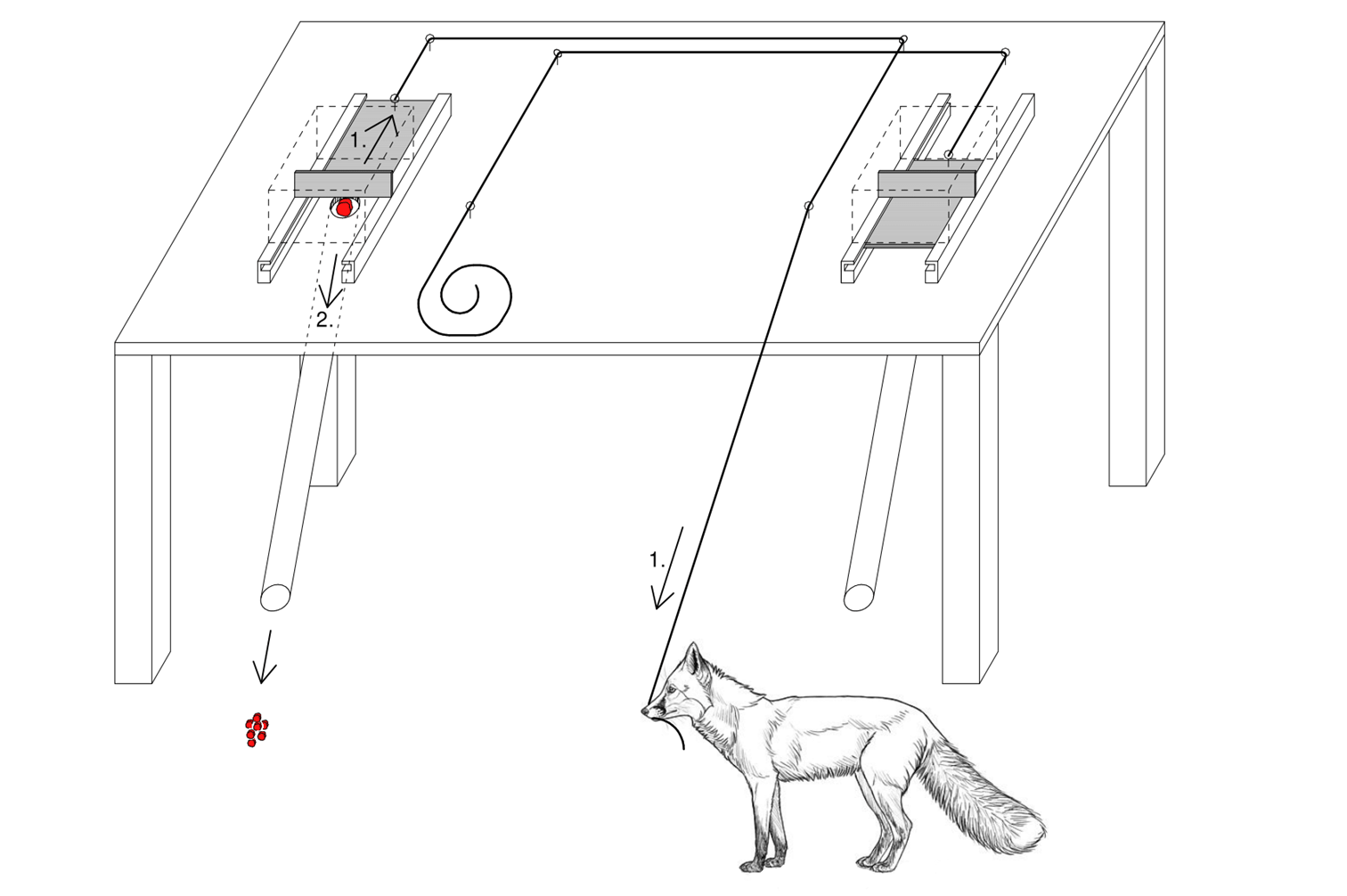

Supplement: Supplementary file 1 [file animals-13-00872-s001.zip › Figure S1.png]

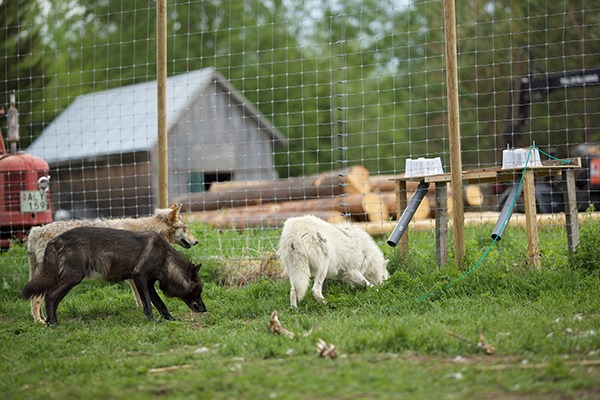

Supplement: Supplementary file 1 [file animals-13-00872-s001.zip › Figure S2.jpg]
